# Randomized study showing the benefit of medical study writing multiple choice questions on their learning

**DOI:** 10.1186/s12909-019-1469-2

**Published:** 2019-01-31

**Authors:** Jose Ignacio Herrero, Felipe Lucena, Jorge Quiroga

**Affiliations:** 1 Department of Internal Medicine, Clínica Universidad de Navarra, Avenida Pío XII, 36, 31008 Pamplona, Spain; 2 Centro de Investigación Biomédica en Red de Enfermedades Hepáticas y Digestivas (CIBERehd), Madrid, Spain; 3 Instituto de Investigación Sanitaria de Navarra (IdiSNA), Pamplona, Spain

**Keywords:** Multiple choice questions, Pathophysiology, General pathology, Exams, Teaching strategies, Gender differences

## Abstract

**Background:**

Writing multiple choice questions may be a valuable tool for medical education. We asked medical students to generate multiple choice questions and studied its effect on their exams. We hypothesized that students generating questions would improve their learning.

**Methods:**

We randomized students in their second and third years at the School of Medicine to write four multiple choice questions on two different sections of General Pathology (Immunopathology and Electrolyte and acid-base status; second year) and Pathophysiology (Blood and Respiratory system; third year). We analyzed whether students writing questions on a section had better results in the exam test in that section than the rest of the students.

**Results:**

Seventy-five (38.2%) students wrote questions for General Pathology and 109 (47.6%) for Pathophysiology. Students that wrote questions obtained significantly better results in the exam than those who did not. In General Pathology, students who wrote questions about Immunopathology obtained better results in that section than those who wrote questions about the other section (5.13 versus 3.86 over 10; *P* = 0.03). In Pathophysiology, the differences between both groups were not significant, but students who wrote good questions about Respiratory system obtained better results in that section than those who wrote good questions about Blood (6.07 versus 4.28 over 10; *P* = 0.015). Male students wrote good questions in Pathophysiology more frequently than female students (28.1% versus 10.4%; *P* = 0.02).

**Conclusions:**

The writing of multiple choice questions by medical students may improve their learning. A gender effect may also influence this intervention. Future investigations should refine its potential role in teaching.

## Background

The construction of questions by the students has been used as a learning tool for medical education. This tool increases the students’ participation in learning and helps them to identify the relevant topics in the lesson content [1, 2]. As designing a good multiple choice question (MCQ) requires a deep knowledge of the material being assessed, it has been suggested that the formulation of MCQ may contribute to a deeper understanding of the topic than other methods [3].

Previous studies have shown that the designing of questions improves their achievement and promotes student motivation [4–6]. On the other hand, other researchers have not found such a beneficial effect [7] or have found only a positive learning effect on certain groups of students [8].

The aim of this study was to investigate whether writing MCQ could improve learning of a topic. The effect of the quality of the questions and of student gender was also evaluated. This was done through a prospective randomized study.

## Methods

### Participants and setting

The study was conducted in the School of Medicine of the Universidad de Navarra, Spain. In our University, Medicine curriculum is studied in six years. Two groups of potential participants were selected: students in their second year studying General Pathology, and students of Pathophysiology (third year). Both are obligatory in the Medicine curriculum. These two subjects are taught by the investigators and by other members of the Department of Internal Medicine. The program of General Pathology is organized in 3 months (January to April) through 48 master classes, including two blocks of 8 classes (Immunopathology and Disturbances of electrolyte and acid-base status), that are taught by two of the investigators (JIH and FL, respectively). Pathophysiology is organized in 8 blocks of 11 master classes. The first four blocks are given in the first three months (September to November). The students have an exam in December that includes these four blocks, and those who reach a qualification of 6 out of 10 don’t need to include these four blocks in the final exam in May. One of the investigators of the study (JIH) teaches two of these four blocks (Blood and Respiratory system pathophysiology).

### Intervention and procedure

One of the investigators (JIH) invited the students to participate in the study in the first lecture of the subject. The students had the opportunity of writing four MCQ with four potential choices. The topic of their questions (Immunopathology or Disturbances of electrolyte and acid-base status for second year, and Blood or Respiratory system pathophysiology for the third year students) was randomly determined, according to the number of their university identity card (even or odds). To stimulate their participation in the study, they could get an extra qualification of up to 0.25 points (out of 10), according to the quality of their questions. The following characteristics were evaluated in each MCQ: importance of the topic, adequately written, unambiguous question (only one valid answer), middle difficulty, and originality. A question was considered to be good if it reached an adequate quality in most of the mentioned characteristics. Students who wrote at least two (of a maximum of four) good questions were analyzed separately. All the students had access to all the questions and their answers (uncorrected by the teacher), independently of whether they had written any question. Two questions of each topic were selected for the exam (with previous changes made by the teacher). The exam of General Pathology included 14 questions of Immunopathology, 14 questions of Disturbances of electrolyte and acid-base status and 62 questions of other topics. Pathophysiology exam included 25 questions of each of the topic explained in the first term (Blood, Respiratory system, Circulatory system and Renal pathophysiology).

### Outcome measure

The outcome measure was the performance of the students in each of the parts of the exam. The effect of writing questions about a topic (and of writing good questions) and gender were studied.

### Statistical analysis

Continuous variables are expressed as median (quartile range), and categorical variables as number (percentage). Differences between groups were compared with Mann-Whitney test (continuous variables) and Chi-square test (categorical variables). Differences were considered to be significant if *P* value was inferior to 0.05. Statistical studies were done with the software Statistical Package for Social Sciences (SPSS). As this was a pilot study and we did not have previous data about the proportion of students’ participation and differences between the groups, sample size had not been previously estimated.

### Ethical considerations

The study was approved by the Universidad de Navarra School of Medicine and by the Committee for Research Ethics of the Universidad de Navarra. Participation of the students in the study was voluntary, without the need of a written informed consent. Assignment of the students to each arm was random. The ethical principles of the World Medical Association Declaration of Helsinki were observed [9].

## Results

### Participation in the study

Seventy-five (38.2%) of the second year students and 109 (47.6%) of the third year students participated in the study. There were no gender differences between participants and non-participants, but participants obtained better results than non-participants, not only in the topics included in the study, but also in other topics of the subject. Third-year students who wrote MCQ also had a better score in General Pathology in the previous year than those who did not (Tables 1 and 2).

**Table 1:** Comparison between students who wrote and who did not write multiple choice questions in General Pathology (second year of Medicine)

	Wrote questions (*N* = 75)	Not wrote questions (*N* = 121)	*P*
Gender			
Male	31 (41.3%)	48 (39.7%)	NS
Female	44 (58.7%)	73 (60.3%)	
Results in the exam^a^			
Immunopathology	4.62 (2.83–5.90)	3.34 (2.06–5.13)	0.007
E & AB^b^	8.10 (7.15–9.05)	6.67 (4.88–8.10)	< 0.001
Other topics	6.44 (5.71–7.18)	5.88 (4.83–6.61)	0.004

^a^Over 10 points

^b^Disorders of electrolyte and acid-base status

**Table 2:** Comparison between students who wrote and who did not write multiple choice questions in Pathophysiology (third year of Medicine)

	Wrote questions (*N* = 109)	Not wrote questions (*N* = 120)	*P*
Results in General Pathology^a^	6.35 (5.26–7.06)	5.67 (4.83–6.52)	0.005
Gender			
Male	32 (29.4%)	41 (34.2%)	NS
Female	77 (70.6%)	79 (65.8%)	
Results in the exam^b^			
Blood	5.34 (3.94–6.80)	4.34 (2.66–6.20)	< 0.001
Respiratory	4.81 (3.47–6.20)	3.74 (2.14–5.47)	< 0.001
Circulatory	4.67 (3.30–5.80)	3.87 (2.14–4.91)	< 0.001
Renal	6.14 (4.80–6.94)	5.05 (3.34–6.40)	< 0.001

^a^In the previous year (106 wrote questions; 112 did not), over 10 points

^b^Over 10 points

### Evaluation of the effect of writing questions about a topic on performance in the exam

Thirty-eight students wrote questions of Immunopathology and 37 wrote questions of disorders of electrolyte and acid-base status. There were no gender differences between them. The performance in Immunopathology of the students who designed MCQ of Immunopathology was significantly better (median qualification: 5.13 versus 3.86 over 10; *P* = 0.03). Other differences between both groups of students were not significant (Table 3). There were not significant differences between male and female students in their perfomance (data not shown).

**Table 3:** Comparison between students who wrote questions about Immunopathology and who wrote questions about disturbances of electrolyte and acid-base status in General Pathology (second year of Medicine)

	Immunopathology	E & BA^a^	*P*
	(*N* = 37)	(*N* = 38)	
Gender			
Male	18 (48.6%)	13 (35.1%)	NS
Female	20 (51.4%)	24 (64.9%)	
Results in the exam^a^			
Immunopathology	5.13 (3.60–6.32)	3.86 (2.19–5.51)	0.03
E & AB^b^	8.10 (6.43–9.05)	8.10 (7.38–9.05)	NS
Other topics	6.61 (5.95–7.18)	6.22 (5.03–7.17)	NS

^a^Over 10 points

^b^Disorders of electrolyte and acid-base status

Fifty-five students wrote questions about blood pathophysiology and 54 about respiratory pathophysiology. There were no gender differences between them. The performance on the four topics included in the exam was not significantly different between both groups (Table 4).

**Table 4:** Comparison between third year students who wrote questions on blood or respiratory pathophysiology

	Blood (*N* = 55)	Respiratory system (*N* = 54)	*P*
Results in General Pathology^a^	6.24 (5.33–7.11)	6.38 (5.08–7.05)	NS
Gender			
Male	18 (32.7%)	14 (25.9%)	NS
Female	37 (67.3%)	40 (70.1%)	
Results in the exam^b^			
Blood	5.60 (4.14–7.07)	5.13 (3.88–6.60)	NS
Respiratory	5.07 (3.47–6.27)	4.54 (3.48–6.17)	NS
Circulatory	4.67 (3.74–5.87)	4.34 (2.34–5.63)	NS
Renal	6.27 (5.07–7.07)	6.07 (4.77–6.83)	NS

^a^In the previous year (54 about blood; 52 wrote questions about respiratory system), over 10 points

^b^Over 10 points

### Evaluation of the quality of the questions

**Fig. 1:**
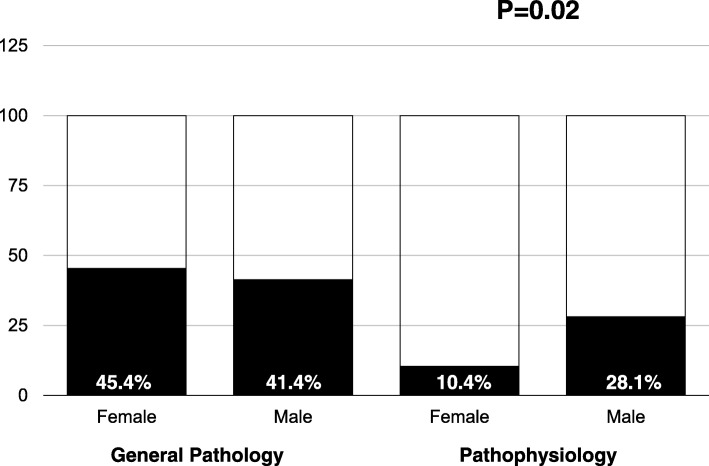
Proportion of medical students who designed good questions in second and third years (General Pathology and Pathophysiology), according to their gender

According to the previously mentioned criteria, 34/75 (45.3%) students of General Pathology wrote at least two questions that could be considered good in most of the criteria: 18/38 (47.3%) in Immunopathology and 16/37 (43.2%) in Disturbances of electrolyte and acid-base status. The proportion of students with to or more good questions in Pathophysiology was 15.6% (17/109): 6/55 (10.9%) in blood and 11/54 (20.4%) in respiratory system. There were no gender differences in General Pathology, but the proportion of male students with good questions in Pathophysiology (28.1%) was significantly higher than female students (10.2%) (Fig. 1).

The students who did good MCQ in Respiratory pathophysiology obtained better results in the exam questions of Respiratory pathophysiology than the students with good questions in Blood pathophysiology (median qualification: 6.07 versus 4.28 over 10; *P* = 0.015). The rest of the comparisons concerning the quality of MCQ were non-significant (Table 5).

**Table 5:** Comparison of the results between students who wrote good questions in General Pathology and Pathophysiology

	Immunology (*N* = 18)	E & BA^a^ (*N* = 16)	*P*
Immunology	5.12 (2.70–7.24)	4.23 (2.51–5.64)	NS
E & BA^a^	8.33 (7.09–9.05)	8.1 (6.79–0.05)	NS
Other topics	7.03 (6.14–7.74)	5.90 (4.75–7.37)	NS
	Blood (*N* = 6)	Respiratory (*N* = 11)	
Blood	6.33 (4.17–7.27)	6.54 (4.94–7.87)	NS
Respiratory	4.28 (2.81–5.48)	6.07 (5.60–7.87)	0.015
Circulatory	4.54 (4.11–6.37)	4.68 (5.47–6.00)	NS
Renal	6.93 (4.91–7.93)	6.80 (6.27–7.87)	NS

^a^Disorders of electrolyte and acid-base status

^b^Over 10 points

## Discussion

The present randomized study reveals that the generation of written MCQ by medical students seems to exert a positive learning effect. Second year students who wrote questions about Immunopathology had better results in this topic that students who did questions in other topic. Furthermore, students who wrote good MCQ about Respiratory Pathophysiology also obtained better results on this topic than those who wrote good questions on other topic.

The positive effect of question designing was not evident in all the comparisons that we made. A possible explanation is the poor quality of the questions designed by our students (less than 50% second-year and less than 20% third-year students wrote at least two good questions). It is likely that the beneficial learning effect of this intervention is evident only if they are good enough. Generating questions by the students stimulate critical thinking and academic performance [10]. The formulation of questions stimulates the students to reflect on their learning progress and start to develop metacognitive capacity [11], but this effect may require a minimal effort.

### Comparison with the literature

Most of the previous studies about the potential effect of question designing are observational studies. Some studies have shown that formulating questions increased the understanding of the topic [8, 12, 13]. The present study reinforces this thought. We have found that question design increase the acquisition of knowledge. However, this is not a universal finding. Other authors have not found that MCQ writing has positive effects on learning [3, 14]. Furthermore, other factors may influence on it.

The quality of the questions is one of these factors [15]. Chin et al. found that basic questions do not help to deep learning of a subject. Our results are in agreement with these findings. On the contrary, Palmer and Devitt [3] did not find a positive effect on the exam results, despite their students made high quality questions. In our study, the students wrote their MCQ shortly before the exams. This last-minute work was probably accompanied by a small effort. Future studies should explore if the inclusion of MCQ design in daily work may increase this effort and improve its learning effect.

Another interesting finding is the difference between genders. Male and female students have different style preferences [16]. Female students usually have a higher degree of genuine motivation (genuine interest in the topic) [17] and males are possibly more stimulated in a competitive environment. Olde Bekknink et al. found that formulating an extra written question had a positive effect on male students [8]. Our study has also found a gender difference. Male students wrote better questions in the Pathophysiology course. Probably, this type of challenge is more motivating for males than for females.

### Strengths and limitations

This was a large, prospective, randomized study that analyzed the potential effect of a MCQ designing on learning. The study was done in two different scenarios (second and third year of Medicine) and with different teachers (two different teachers in second year and the same teacher in the third year). This intervention is not time-consuming. Thus, it is easy to apply in large groups.

A major limitation was the fact of the poor quality of the MCQ formulated by the majority of the students. Probably, the students’ effort for the generation of questions was small and the objective of a deep learning was not obtained in many of them. Furthermore, the classification of the MCQ as good depended of the subjective qualification of the investigators (according to pre-specified criteria). The use of more objective criteria would have been desirable. Another limitation is the absence of a universal demonstration of the beneficial effect of MCQ generation. The only significant differences that were found suggested that students who designed MCQ on a topic obtain a better score in this topic, but this finding was not confirmed in all the comparisons.

## Conclusions

Formulating MCQ by students seems to exert a positive learning effect. This effect seems to be greater in male students and may be restricted to students who make a significant effort that allow them to formulate good questions. Future research may refine this strategy of participating of students in their learning.

## Abbreviations

E&AB: Disturbances of electrolyte and acid-base status
MCQ: Multiple choice questions
