# Correction to: Randomized study showing the benefit of medical students writing multiple choice questions on their learning

**DOI:** 10.1186/s12909-019-1506-1

**Published:** 2019-03-04

**Authors:** Jose Ignacio Herrero, Felipe Lucena, Jorge Quiroga

**Affiliations:** 1 Department of Internal Medicine, Clínica Universidad de Navarra, Avenida Pío XII, 36, 31, 008 Pamplona, Spain; 2 Centro de Investigación Biomédica en Red de Enfermedades Hepáticas y Digestivas (CIBERehd), Madrid, Spain; 3 Instituto de Investigación Sanitaria de Navarra (IdiSNA), Pamplona, Spain

**Correction to: BMC Medical Education (2019) 19:42**

**https://doi.org/10.1186/s12909-019-1469-2**

Following publication of the original article [1], the author reported an error in the title; the word study should be changed to students as indicated below:

Incorrect title in original article: Randomized study showing the benefit of medical study writing multiple choice questions on their learning.

Correct title: Randomized study showing the benefit of medical students writing multiple choice questions on their learning.

## References

[1] Herrero, et al. Randomized study showing the benefit of medical students writing multiple choice questions on their learning. BMC Medical Education. 2019;19(42). 10.1186/s12909-019-1469-2. 10.1186/s12909-019-1506-1 PMC6399937 30832650

